# Draft whole-genome sequence of *Pediococcus pentosaceus* GS4, a potential probiotic strain isolated from Indian fermented food

**DOI:** 10.1128/mra.00620-24

**Published:** 2025-09-25

**Authors:** Suzana Ghosh, Asit Ranjan Ghosh

**Affiliations:** 1 Department of Integrative Biology, School of Bioscience and Technology (SBST), Vellore Institute of Technology (VIT) 728495 , Vellore, Tamil Nadu, India; University of Strathclyde, Glasgow, United Kingdom

**Keywords:** probiotics, genome analysis

## Abstract

*Pediococcus pentosaceus* GS4 is a gram-positive potential probiotic strain isolated from Indian food, Khadi. The probiotic potential of GS4 is evidenced by published research works *in vitro* and in mice. Here, we report the 1.6 Mb assembly and annotation of the whole-genome sequence of *P. pentosaceus* GS4.

## ANNOUNCEMENT

Probiotics are microbes that, on adequate consumption, provide benefits to human health (1). This manuscript presents the genome of potential probiotic strain *Pediococcus pentosaceus* GS4, a gram-positive tetrad, isolated from home-made Indian fermented food, Khadi, in 2010 (2). Formerly, a Khadi sample was collected aseptically from South India (12.93 N, 79.14 E) and was processed within 2 h of collection. Khadi (0.1 g) was serially diluted, and 50 µL of diluted samples was plated onto de Man Rogosa Sharpe (MRS) agar (Himedia, India) and incubated at 37°C for 24 h. Following, a selected isolated colony was inoculated in an MRS broth (pH 6.5 ± 0.2) and was incubated at 37°C for 24 h aerobically. The same broth was re-streaked to obtain pure colonies. Obtained colonies were then tested *in vitro* and *in vivo* to examine the probiotic potential, as evidenced by previously published research works. Those studies showed that GS4 tolerates acid and bile salt; exhibits anti-oxidative (3), antimicrobial (4), and cell adherent (5) properties; biohydrogenates essential fatty acid (6); assimilates cholesterol (2); induces apoptosis in cell line (7); and mitigates azoxymethane-induced colon cancer and cadmium-induced toxicity in mice (8, 9). Strain GS4 was previously identified as *P. pentosaceus* based on 16S rRNA sequencing, and the sequence was deposited in GenBank with the accession number HM044322 (2).

GS4 was preserved by lyophilization. For isolation of genomic DNA (gDNA), lyophilized GS4 was sub-cultured aerobically onto MRS agar, and an isolated single colony was grown in the MRS broth for 24 h at 37°C. This culture was subjected to gDNA extraction using PureLink™ Genomic DNA Mini Kit (Thermo Fisher Scientific, USA). The purity of extracted gDNA was evaluated in the CLARIOstar plus model multimode reader (BMG Labtech) using a Lvis plate. Library preparation was performed using the Ion Xpress Plus Fragment Library Kit (Thermo Fisher Scientific, USA). Sequencing was conducted on the Ion Torrent S5 system (Thermo Fisher Scientific, USA), using a 530 chip. Sequencing yielded a total of 5,425,637 reads with an average read length of 190 bp. Raw sequencing reads were subjected to quality control analysis using the FastQC v0.115 tool (10). Trimming and adapter removal were performed using the inbuilt Torrent Suite™ Software 5.12. These quality-filtered (processed) reads were used for *de novo* genome assembly in SPAdes v. 2.3 (11) using the “careful” option for mismatch correction. After the removal of small scaffolds (<500  bp), a total of 114 contigs were obtained. The exact N50 value was 19,789  bp, and the sequencing read coverage calculated from total bases sequenced over the assembled genome size was approximately 627×. In all, 1,643,895  bp (1.6  Mb) of assembled consensus sequences were obtained with 37.11% of guanine, cytosine (GC) (Table 1). Functional annotation of the genome was performed using the NCBI Prokaryotic Genome Annotation Pipeline (PGAP), version 6.7 (12). The genome has 65.36% completeness, as appeared in CheckM analysis (v1.2.2) (13). The total number of coding sequences was 1,788, and the total genes were 1,816. The number of identified frameshifted proteins was 379. Calculated Average Nucleotide Identity (ANI) value was 99.57% while comparing the genome of GS4 and *P. pentosaceus* MIANGUAN (accession NZ_CP156667.1) using EzBioCloud ANI calculator and OrthoANI algorithm (14) with default parameters. For all tools other than where mentioned, default settings were applied.

**TABLE 1:** Features of annotated whole-genome sequence of *P. pentosaceus* GS4

Strain	Raw reads	Genome size (bp)	Contig N50	Contig L50	No. of predicted protein coding gene	No. of contigs
*P. pentosaceus* GS4	5,425,637	1,643,895	19,789	27	37.11	114

## Data Availability

The genome information is available under the NCBI BioProject accession number PRJNA1114943. The *Pediococcus pentosaceus* GS4 whole-genome shotgun sequencing project has been deposited in DDBJ/ENA/GenBank under the accession number JBDQYX000000000.1. Version described in this paper is JBDQYX000000000.1. Raw reads are available under the SRA accession number SRR29303853.
